# Dissection of the radical reactions linked to fetal hemoglobin reveals enhanced pseudoperoxidase activity

**DOI:** 10.3389/fphys.2015.00039

**Published:** 2015-02-20

**Authors:** Khuanpiroon Ratanasopa, Michael Brad Strader, Abdu I. Alayash, Leif Bulow

**Affiliations:** ^1^Pure and Applied Biochemistry, Department of Chemistry, Lund UniversityLund, Sweden; ^2^Laboratory of Biochemistry and Vascular Biology, Center for Biologics Evaluation and Research, Food and Drug AdministrationSilver Spring, MD, USA

**Keywords:** hemoglobins, fetal blood, peroxidases, kinetics, spectrophotometry, quantitative mass spectrometry

## Abstract

In the presence of excess hydrogen peroxide (H_2_O_2_), ferrous (Fe^+2^) human hemoglobin (Hb) (α2β2) undergoes a rapid conversion to a higher oxidation ferryl state (Fe^+4^) which rapidly autoreduces back to the ferric form (Fe^+3^) as H_2_O_2_ is consumed in the reaction. In the presence of additional H_2_O_2_ the ferric state can form both ferryl Hb and an associated protein radical in a pseudoperoxidative cycle that results in the loss of radicals and heme degradation. We examined whether adult HbA (β2α2) exhibits a different pseudoenzymatic activity than fetal Hb (γ2α2) due to the switch of γ to β subunits. Rapid mixing of the ferric forms of both proteins with excess H_2_O_2_ resulted in biphasic kinetic time courses that can be assigned to γ/β and α, respectively. Although there was a 1.5 fold increase in the fast reacting γ /β subunits the slower reacting phases (attributed to α subunits of both proteins) were essentially the same. However, the rate constant for the auto-reduction of ferryl back to ferric for both proteins was found to be 76% higher for HbF than HbA and in the presence of the mild reducing agent, ascorbate there was a 3-fold higher reduction rate in ferryl HbF as opposed to ferryl HbA. Using quantitative mass spectrometry in the presence of H_2_O_2_ we found oxidized γ/β Cys93, to be more abundantly present in HbA than HbF, whereas higher levels of nitrated β Tyr35 containing peptides were found in HbA samples treated with nitrite. The extraordinary stability of HbF reported here may explain the evolutionary advantage this protein may confer onto co-inherited hemoglobinopathies and can also be utilized in the engineering of oxidatively stable Hb-based oxygen carriers.

## Introduction

Fetal hemoglobin (HbF) is the main oxygen carrier protein in the human fetus during the last 7 months of development in the uterus and remains the dominating Hb in the newborn until the age of approximately 6 months. In contrast to the adult form (HbA), which has a quaternary α_2_β_2_ structure, HbF is composed of two alpha and two gamma chains, commonly denoted as α_2_γ_2_. Even though the overall structure shows strong similarities with that of HbA, the two Hbs exhibit some important differences in their biophysical properties, such as O_2_ binding affinity (Hofmann and Brittain, 1996); and binding kinetics to other ligands (Engel et al., 1969; Manca and Masala, 2008). In a healthy adult, HbF levels are very low, less than 0.6%, but can be elevated in pregnant women. HbF levels are also enhanced under some specific conditions, notably in β-thalassemia, hereditary persistence of fetal hemoglobin (HPFH) and sickle cell anemia (SCD) (Forget, 1998; Olsson et al., 2010). Due to its ability to solubilize HbS polymers, switching the synthesis of HbF within affected patients RBCs has been shown to have an anti-sickling therapeutic potential in sickle cell disease (SCD). Moreover, a recent study demonstrates that an increase of HbF levels in sickle cell anemia patients helps to reduce kidney damages (Risso et al., 2012). Therefore, several efforts are currently (Reeder, 2010; Akinsheye et al., 2011) being undertaken to identify new active substances that can induce HbF in SCD or β-thalassemia patients (Bianci et al., 2007). However, HbF levels can be elevated also under other and more normal conditions, e.g., in adults that have been exposed to high altitude hypoxia often exhibit higher expression levels (Lebensburger et al., 2011).

Hb becomes toxic if it is released from erythrocytes. This toxicity is largely caused by oxidative reactions linked to the Hb molecule, which in turn results in damage to surrounding tissues, proteins, nucleic acids and lipids (Alayash, 2004; Bianci et al., 2007). Due to the structural differences between HbA and HbF, the kinetics of the radical reactions associated with these globins is dissimilar. For instance, HbF has been proposed to be one of the main causative agents behind the inflammatory damage of the placenta in preeclampsia (Olsson et al., 2010). HbF has therefore been suggested to be, either alone or together with alpha-1-microglobulin (A1M), most valuable as a biomarker for prediction of preeclampsia (Anderson et al., 2012). It is therefore, essential to develop a better understanding of the radical reactions associated particularly with HbF in order to design against or for these properties.

It is well known that cell-free Hb molecules are susceptible to and often are involved in various oxidative reactions, for instance, when Hb is exposed to H_2_O_2_–rich environments (Kvist et al., 2007; Olsson et al., 2012). The reaction between Hb and H_2_O_2_ may lead to an irreversible loss of Hb activity, and ferryl Hb which is formed as an intermediate, is a highly reactive species which together with other Hb oxidation products (i.e., heme) may contribute to inflammatory responses (Baek et al., 2012; Belcher et al., 2014). The reaction between ferrous Hb and H_2_O_2_ results in ferryl heme (Fe^4+^) formation without generating a protein based free radical. The presence of the highly oxidizing ferryl heme species may ultimately lead to heme degradation and heme-protein crosslinking products (Reeder et al., 2008a; Alayash, 2014). On the other hand, the reaction with ferric Hb results in oxoferryl (Fe^4+^) and protein– or prosthetic group associated free radicals. The oxyferryl may also react further with excess H_2_O_2_, yielding ferric Hb and H_2_O (Reeder et al., 2008a). This activity is referred to as “pseudoperoxidative” because the Hb is unable to harness its radicals like other classical oxidases, such as cytochrome oxidase, and prostacyclin synthases (Stubbe and Riggs-Gelasco, 1998). Therefore, the reaction involving ferric Hb can act as a competitor to the reaction between oxyHb and H_2_O_2_ to limit the levels of heme degradation products as well as to reduce the maximal levels of ferryl Hb (Reeder et al., 2008a). Moreover, investigation of the contribution of individual Hb subunits to the overall redox activity of Hb and their relative stabilities under oxidative stress revealed that α unlike β and γ are able to reduce ferryl Hb possibly through an internal electron pathway, involving Tyr42 (Reeder et al., 2008a; Mollan et al., 2013).

In this study, the pseudoperoxidative activity of HbF was compared with that of HbA. Hb can undergo a range of oxidative reactions. In order to facilitate the interpretation and analysis of the obtained experimental data, the main focus was put on the ferric form of Hb We show that ferric HbF can react rapidly with H_2_O_2_ to exert a faster association rate constant relative to HbA. However, both the autoreduction and ascorbate mediated reduction rates were faster for HbF. Kinetics of ferryl reduction by ascorbate demonstrated that HbF has a lower *K_D_* value for the high affinity pathway. The faster turnover rate of HbF is also demonstrated by the reaction between oxyHb and nitrite. To expand on these studies, we performed a mass spectrometry characterization to elucidate how different residues in the α, β, and γ globins respond to H_2_O_2_ exposure. By combining the experimental results with existing structural information, an anti-oxidative role of HbF can be envisaged *in vivo*. Immediate implications in clinical settings as well as in the design of functional and stable Hb-based oxygen carriers (HBOCs) can be anticipated (Alayash, 2014).

## Materials and methods

### Materials

HbA and HbF purified from healthy volunteers and cord blood, respectively, were kindly provided by Prof Bo Akerstrom at the Biomedical Center, Lund University. Both the oxy and the ferric forms of the proteins were examined. Ferric Hb was prepared by adding 1.5 M excess of potassium ferricyanide K_3_[Fe(CN)_6_]. The sample was incubated for 5 min under visible light and excess of ferri-ferrocyanide was then removed by filtration on a Sephadex G-25 column. All experiments were performed under aerobic conditions at 25°C, if not otherwise specified.

### Autooxidation

Oxyhemoglobin concentrations were measured spectrophotometrically at 523 nm using 7.12 mM^−1^ cm^−1^ as molar extinction coefficient (Snell and Marini, 1988; Vandegriff et al., 2006). The autoxidation experiments were performed in 0.1 M sodium phosphate buffer pH 7.4 by monitoring the decrease of oxyHb over 48 h. The autoxidation rate constants were obtained by fitting to a first order exponential equation (Strader et al., 2014).

### Oxidation reactions of ferric hemoglobin with hydrogen peroxide

The oxidation of ferric Hb was monitored by a stopped-flow rapid mixing approach. The reaction between ferric Hb and H_2_O_2_ was thus performed under pseudo first order reaction conditions by placing 20 μM of ferric Hbs and H_2_O_2_ at concentrations up to 1000 μM in separate syringes. The two components were rapidly mixed and the time course of reaction was followed at 405 nm using the RX-2000 rapid kinetic accessory (Applied Photophysics Limited, United Kingdom). The time course was fitted to a double exponential equation. The rate constant of each reaction phase could then be obtained by linear regression and was plotted as a function of the hydrogen peroxide concentration.

### Ferryl hemoglobin reduction by ascorbate

The ferryl Hb reduction study was performed according to a previously described method with slight modifications (Reeder et al., 2008a). Briefly, 20 μM ferric Hb was placed in a cuvette, supplemented with 100 μM H_2_O_2_to convert the globin to the ferryl form. 10 nM of catalase was added to stop the reaction. Increasing concentrations of ascorbate were then added (0–500 μM), and the formation of ferric Hb was monitored spectrophotometrically until the reaction was completed using an Agilent 8453 instrument. The time course of reaction at 405 nm was fitted to a double exponential equation using the Microsoft Excel Solver program. The set of obtained rate constants was then plotted vs. the ascorbate concentration, and the data were fitted to a double rectangular hyperpolar function (Reeder et al., 2008b).

### Liposome oxidation

One micro molar of ferric Hb in 20 mM sodium phosphate buffer pH 7.4 was incubated together with liposomes prepared by sonicating 23% phosphatidylcholine in the same buffer. The final concentration of liposome was 200 μM. The formation of conjugated dienes was monitored over time at 234 nm using ε_234_ = 2.5 × 10^4^ M^−1^cm^−1^ (Egmond et al., 1976).

### Oxyhemoglobin and nitrite

Forty micro molar of oxyHb in 20 mM sodium phosphate buffer pH 7.4 was rapidly mixed with a 1.0 mM sodium nitrite solution in the stopped-flow using a RX-2000 rapid kinetic accessory provided by the manufacturer. Absorption spectra ranging from 450 to 700 nm were recorded every 15 s for 5 min with a scanning rate of 2880 nm/min and 3 nm interval. The multispectra obtained were then analyzed by Convex Constraint Analysis program (CCA plus) (Perczel et al., 1991). By using default setting parameters, the reaction product components over the time were obtained.

### Comparative analysis of oxidative hotspots in HbA and HbF using quantitative proteomics

The effects of H_2_O_2_ and NaNO_2_-mediated oxidation of key amino acids in “hotspots” within γ/β and α subunits were investigated in both proteins. by a proteomic profiling study (Alayash, 2004; Jia et al., 2007; Pimenova et al., 2010). HbA and HbF stocks were treated with excess K_3_[Fe(CN)_6_] to generate the ferric form of both proteins. Removal of K_3_[Fe(CN)_6_] was accomplished using a G-25 Sephadex (Sigma) column. In one set of reactions, 10 μM ferric HbA and HbF samples were treated with incremental doses (0, 100 μM, 200 μM, 300 μM, 400 μM, and 500 μM) of H_2_O_2_. In a second set of reactions, 40 μM ferrous HbA and HbF was treated with 1 mM NaNO_2_. Both experimental sets were incubated overnight in 20 mM sodium phosphate buffer pH 7.4. All samples were processed, trypsinized and analyzed (in triplicate) by reverse phase liquid chromatography tandem mass spectrometry (RP LC/MS/MS) using an Easy nLC II Proxeon nanoflow HPLC system coupled online to a Q-Exactive Orbitrap mass spectrometer (Thermo Scientific) as previously described (Strader et al., 2014). Briefly, data were acquired using a top10 method (for 60 min) dynamically choosing the most abundant precursors (scanned at 400–2000 m/z) from the survey scans for HCD fragmentation. The database search engine Mascot 2.4 (Matrix Sciences, London, UK) was utilized to identify oxidized version of “hotspot” peptides, by searching all MS/MS data against the Swiss-Prot Human database (release 2014_03; contains 542782 sequence entries) supplemented with porcine trypsin using the differential search parameters specified for detecting variable modifications including oxidation of methionine (+16 daltons), cysteine (+32 and +48 daltons) and nitration of tyrosine (+45 daltons). Because all experimental samples were denatured and treated with iodoacetamide prior to trypsinization, an additional static search involving carbamidomethylation of cysteine was included to identify all unoxidized cysteine (not oxidized in presence of H_2_O_2_). The precursor ion mass tolerance was ± 10 ppm and the fragment ion mass tolerance was ± 0.025 Da. Mascot output files were analyzed using the software Scaffold 4.2.0 (Proteome Software Inc.) Scaffold filters were adjusted to only include peptide identifications that were accepted if they could be established at greater than 99.0% probability by the Peptide Prophet algorithm (Keller et al., 2002). This resulted in a false positive discover rate (FDR) of 0.1%. Protein identifications were accepted if they could be established at greater than 95.0% probability and contained at least two identified peptides. Protein probabilities were assigned by the Protein Prophet algorithm (Nesvizhskii et al., 2003). Extracted ion chromatograms of the modified (and unmodified) version of tryptic peptides listed in Table 1 were used to quantify differences between HbA and HbF. For relative quantification, the ratio of each oxidized “hot spot” peptide was calculated based on the sum of the extracted ion chromatogram (XIC) peak area of all forms (oxidized and unmodified) to be 100%. Hotspot residues used for comparisons included conserved residues found on β, γ, and α subunits (Jia et al., 2007).

**Table 1:** **“Hotspot” modified amino acids identified in the reaction of hemoglobins with peroxide**.

Peptides^*^	**Modified residue**	**Modification**	**(+) Charge State**	**m/z**
**^83^GTFATLSELHCDK^95^**	**Cys93(β)**	**oxidation (+48)**	**2**	**735.33**
**^83^GTFAQLSELHCDK^95^**	**Cys93(γ)**	**oxidation (+48)**	**2**	**748.84**
**^83^GTFATLSELHCDKLHVDPENFR^104^**	**Cys93(β)**	**oxidation (+48)**	**3**	**860.41**
			**4**	**645.31**
**^83^GTFAQLSELHCDKLHVDPENFK^104^**	**Cys93(γ)**	**oxidation (+48)**	**4**	**645.06**
^106^LLGNVLVCVLAHHFGK^121^	Cys112(β)	oxidation (+48)	3	589.99
			2	884.48
**^100^LLSHCLLVTLAAHLPAEFTPAVHASLDK^127^**	**Cys104(α)**	**oxidation (+48)**	**4**	**754.91**
^41^MFLSFPTTK^59^	Met32(α)	oxidation (+16)	2	544.27
			2	1087.54
^41^FFESFGDLSTPDAVMGNPK^59^	Met55(β)	oxidation (+16)	3	692.32
			2	1037.97
^41^FFDSFGNLSSASAIMGNPK^59^	Met55(γ)	oxidation (+16)	3	669.32
			2	1003.47
**^17^VGAHAGEYGAEALER^31^**	**Tyr24(α)**	**Nitration (+45)**	**3**	**525.58**
^41^TYFPHFDLSHGSAQVK^56^	Tyr42(α)	Nitration (+45)	3	626.97
			4	470.48
**^31^LLVVYPWTQR^40^**	**Tyr35(β and γ)**	**Nitration (+45)**	**2**	**660.36**

* *Bolded sequences correspond to those peptides with amino acids that are conserved among subunits and/or where significance differences in oxidation were observed*.

## Results

**Figure 1:**
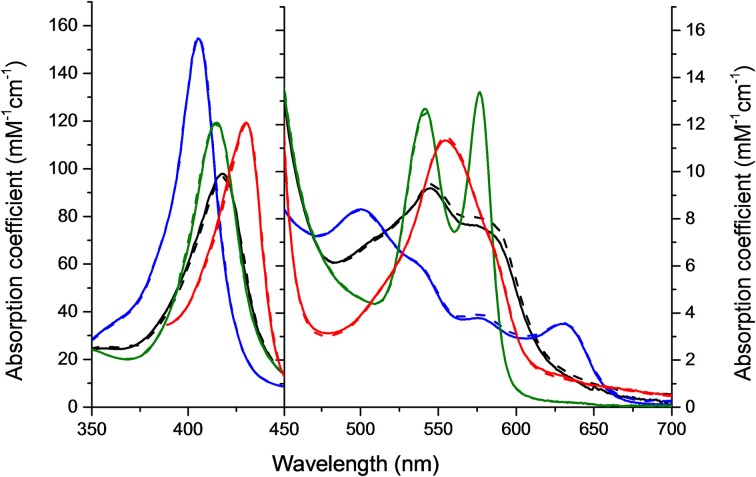
**Molar absorption coefficients of the four different Hb forms used in 0.1 mM sodium phosphate buffer pH 7.4 at 25°C**. Green, oxy; Red, deoxy; Blue, ferric; and Black, ferryl form. Solid lines represent HbF and dashed lines HbA.

**Figure 2:**
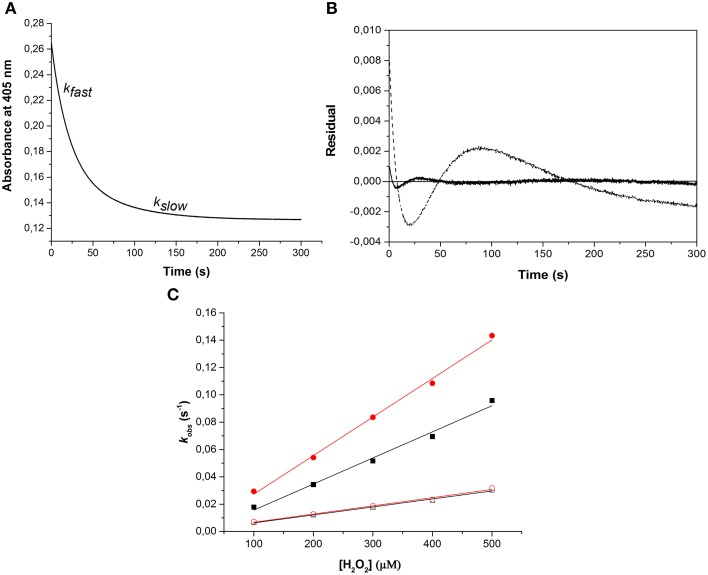
**Oxidation of ferric hemoglobin by hydrogen peroxide**. The experiment was carried out under pseudo first order reaction conditions, where the concentration of Hb was kept constant at 10 μM while varying the concentration of H_2_O_2_ (100–500 μM). **(A)** Represents an example of the time course for the reaction using 10 μM ferric HbA and 500 μM hydrogen peroxide monitored at 405 nm. The time course was best fitted to a double exponential equation giving two rate constants, k_fast_ and k_slow_, respectively. **(B)** Residuals from the fit of the time course to a single exponential (dashed line) and a double exponential (solid line) equation. **(C)** Shows plots of k_obs_ against H_2_O_2_ concentration used. (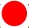): k_fast_ HbF, (■): k_fast_ HbA, (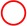): k_slow_ HbF, and (☐): k_slow_ HbA. Solid lines represent the linear regression function.

HbA and HbF in the oxy-, deoxy-, ferric and ferryl forms all showed identical and typical absorption spectra associated with hemoglobins in the range 350–700 nm. For instance, for the oxyHbs a soret peak was easily identified at 415 nm and Q bands at 540 and 577 nm, respectively (Figure 1). Cell-free Hb is rapidly oxidized outside the protective environment of the red blood cells. The autooxidation measurements were therefore directly comparable for the two hemoglobins and they exhibited similar autoxidation rates of 0.046 h^−1^ and 0.049 h^−1^ for HbA and HbF, respectively. These values are close to reported autooxidation rates for human hemoglobins (Strader et al., 2014). To determine the oxidation rate of ferric Hb by H_2_O_2_, a set of experiments was carried out under pseudo first order reaction conditions. The time course of the drop in ferric Hb levels at 405 nm fitted best with a double exponential equation. By this fitting method, two rate constants were obtained, and assigned as *k_fast_* and *k_slow_*, respectively. The set of rate constants was then plotted against the concentration of H_2_O_2_ resulting in a linear relationship as shown in Figure 2. The slope of the plot gives a second-order rate constant for the reaction between ferric Hb and H_2_O_2_. The k_fast_ of the reaction was found to be = 1.89 × 10^−4^ ± 0.01 × 10^−4^ μM^−1^s^−1^ and 2.82 × 10^−4^ ± 0.03 × 10^−4^ μM^−1^s^−1^ for HbA and HbF, respectively. The k_slow_ of the reaction was found to be very similar for both hemoglobins, 5.72 × 10^−5^ ± 0.12 × 10^−5^ μM^−1^s^−1^ and 6.08 × 10^−5^ ± 0.08 × 10^−5^ μM^−1^s^−1^for HbA and HbF, respectively. These results can be assigned to the difference of oxidation between α and β/γ chains. The oxidation rate of the γ chain is thus 50% higher than the beta chain.

**Figure 3:**
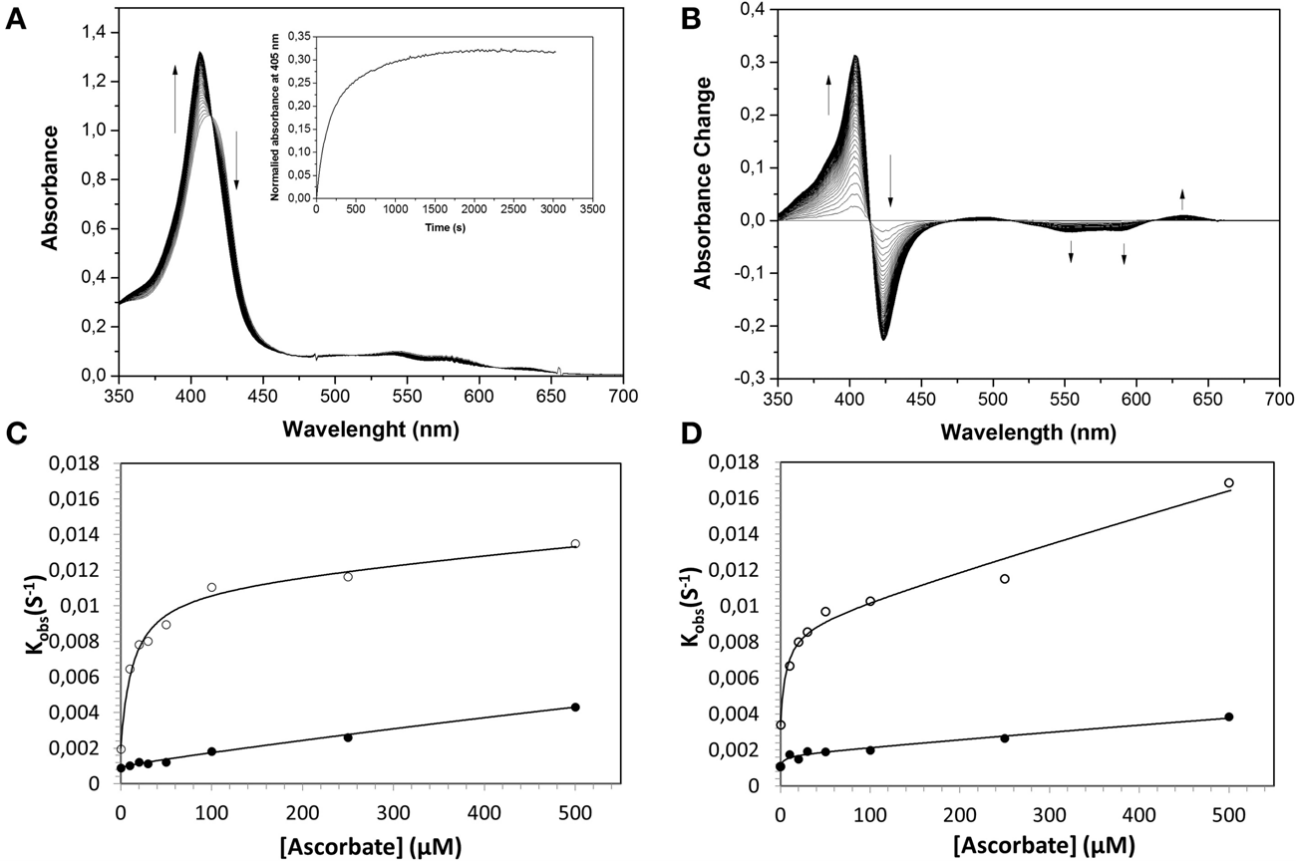
**Reduction of ferrylHb by ascorbate, illustrating the influence of increasing ascorbate concentrations on the reaction rate constants**. Ferryl HbF (10 μM) was generated by adding hydrogen peroxide to ferric Hb at pH 7.4. Catalase was then supplemented to remove the excess of hydrogen peroxide and ascorbate was added to a final concentration of 100 μM. **(A)** Represents the absorption spectra monitored every 15 s over 25 min. *Inset*: The time course of reaction at 405 nm. **(B)** Shows spectra taken from **(A)** where the starting ferryl spectra have been set to zero. The rate constants for the two phases were determined by fitting the reaction time courses to a double exponential equation. **(C)**: HbA, **(D)**: HbF. The solid lines represent the double rectangular hyperbolic functions. Open symbols represent Hb α subunit, and closed symbols represent Hb β subunit (or γ subunit in HbF).

**Figure 4:**
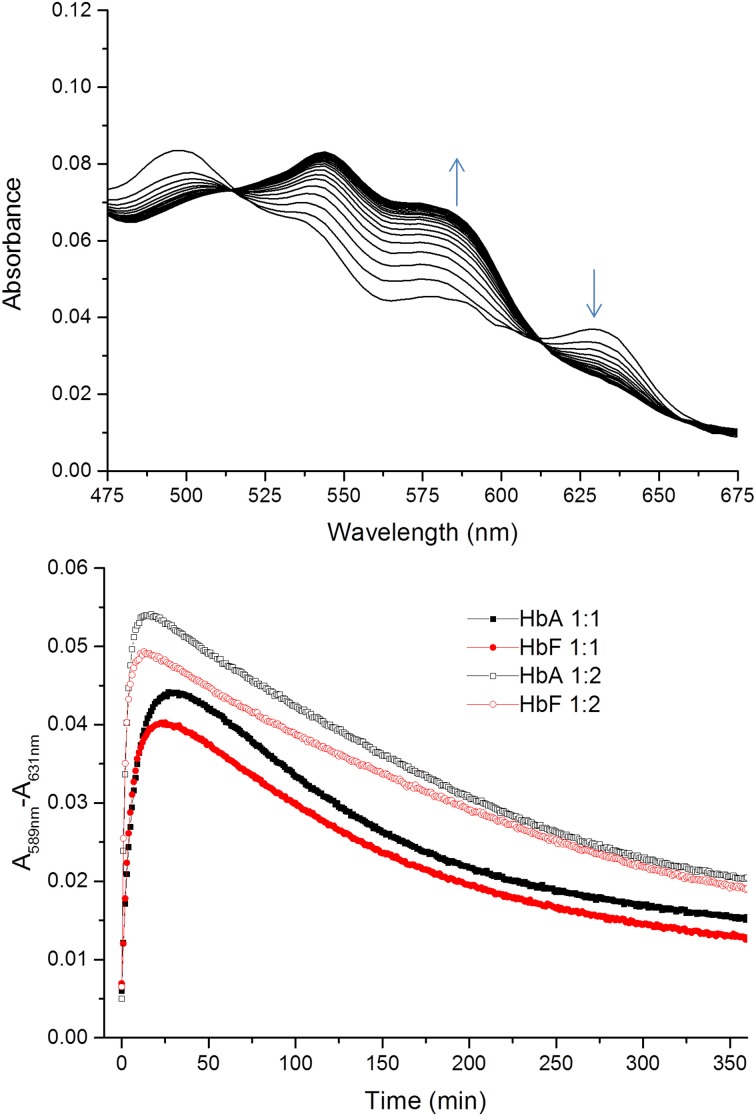
**Time courses for the reaction between ferric Hb and hydrogen peroxide**. The spectra of the reactions were recorded every 1 min for 360 min within the 450–700 nm region. The upper figure shows a typical spectrum during the first 20 min of the reaction between ferric HbF and hydrogen peroxide. The appearance of ferryl Hb species (589 nm) and the decrease of ferric species (631 nm) are indicated with arrows. The lower part illustrates the differences in absorbance at 589–631 nm over time for the reaction between ferric Hb and hydrogen peroxide. Closed symbols represent the reaction when the Hb:hydrogen ratio is 1:1 and open symbols 1:2. Black and red represent the reactions of HbA and HbF, respectively.

Similarly, the rate of ferryl Hb reduction was determined by adding a mild reducing agent, ascorbate (Figure 3). As can be seen in this figure, a biphasic time course can be clearly distinguished. For the fast rate constant, both hemoglobins exhibited a double rectangular hyperbolic behavior dependent on the concentration of ascorbate. This reaction profile has previously been reported for native HbA and some hemoglobin mutants (Reeder et al., 2008a). However, the slow rate constant displayed no sign of having double rectangular hyperbolic character. The fast rate constant increased from 1.94 × 10^−3^ s^−1^ to 1.35 × 10^−2^ s^−1^ when the concentration of ascorbate was increased in the case of HbA. A similar trend was observed for HbF. The fast rate constant increased from 3.40 × 10^−3^ s^−1^ to 1.69 × 10^−2^ s^−1^ upon incremental additions of ascorbate from 0 to 500 μM. A difference in the high affinity pathway between these two hemoglobins can however, be observed; HbF has a lower *K_D_* value (6 μM) than HbA (*K_D_* = 13 μM). The auto-reduction rate constants, obtained when no ascorbate was added, were found to be 76% higher for HbF than HbA, 3.41 × 10^−3^ s^−^1 and 1.94 × 10^−3^ s^−1^, respectively. The complete conversion of first oxidizing ferric Hb to the ferryl form with a low concentration of H_2_O_2_, followed by its auto-reduction is shown in Figure 4. Higher ferryl Hb levels are found for HbA at both ratios of heme to H_2_O_2_ tested, 1:1 and 1:2.

As described previously (Reeder et al., 2008a,b), the reduction of ferryl Hb embraces two alternative mechanisms, a high affinity pathway involving “a through-protein electron hopping mechanism” and a low affinity site involving a direct reduction of the ferryl iron, where ascorbate directly accesses the heme pocket. Since HbA and HbF carry the same α chains and since there are strong similarities between the β and γ chains, the reaction mechanisms are most likely analogous. However, when considering the low affinity site of the α chain reaction, it is obvious that there is a strong influence of the β and γ chains in the reduction pathway. The *K_D_* (estimated by fitting) at this site was found to be 6 and 10 mM for HbA and HbF, respectively. Similarly, the *k_max_* was found to be 0.03 s^−1^ and 0.16 s^−1^ for HbA and HbF, giving corresponding *k_max_*/*K_D_* values of 0.005 and 0.016 s^−1^mM^−1^. This implies that the efficiency of reduction using ascorbate is 3-fold higher for HbF compared to HbA. This in turn indicates that the heme pocket in HbF is most likely more accessible for ascorbate than the one in HbA.

Phosphatidylcholine liposomes were used as a model for studying the ability of Hb to induce lipid peroxidation. Ferrous and ferric Hb do not react with lipids, however, a lipid hydroperoxide (LOOH), which is present in trace amounts in membranes or in a liposome preparation step can react. The reaction between ferric Hb (HbFe^3+^) and a lipid hydroperoxide (LOOH), yields ferryl Hb (HbFe^4+^) and a lipid alkoxyl radical (LO•) in the first step. The newly formed ferryl Hb can then be reduced back to ferric Hb by removal of hydrogen either from lipid (LH) or LOOH and a lipid alkyl radical (L•) or lipid peroxyl radical (LOO•) is formed. In the presence of oxygen, the lipid alkyl radical will rapidly react to form a lipid peroxyl radical. The lipid oxidation started by ferric Hb is initially slow. However, when ferryl Hb and radical concentrations reach a critical point, a cascade of lipid peroxidation is started. This results in a rapid increase in the levels of lipid based conjugated dienes which can be followed spectrophotometrically at 234 nm. Therefore, the time course of reaction is composed of a lag phase and a propagational part. The overall lipid oxidation cascade is presented in Equations (1–5).

(1) $$\begin{array}{l} {\text{HbFe}}^{3+}+\text{ LOOH }\to {\text{HbFe}}^{4+}-\text{OH}-\;+\text{ LO}•\\ \;\left(\text{starting reaction},\text{ lag phase}\right) \end{array}$$

(2) $$\begin{array}{l} {\text{HbFe}}^{4+}-\text{OH}-\;+\text{ LH}\to \;{\text{HbFe}}^{3+}-{\text{H}}_{2}\text{O}+\text{L}•\\ \;\left(\text{propagational phase}\right) \end{array}$$

(3) $$\begin{array}{l} {\text{HbFe}}^{4+}-\text{OH}-\;+\text{ LOOH}\to \;{\text{HbFe}}^{3+}+\text{ LOO}•+\;{\text{H}}_{2}\text{O}\\ \;\left(\text{propagational phase}\right) \end{array}$$

(4) $$\text{L}•+\;{\text{O}}_{2}\to \text{LOO}•$$

(5) LH+ LOO• →L•+ LOOH (propagational phase)

**Figure 5:**
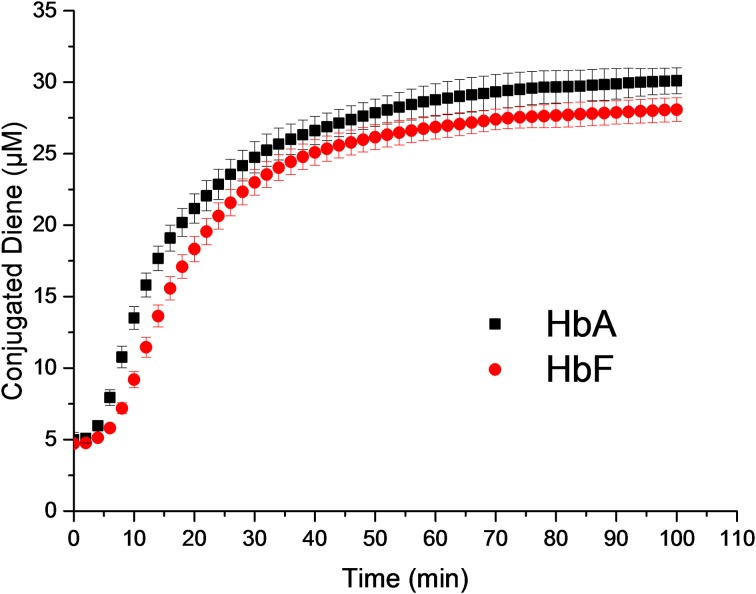
**Liposome oxidation by Hb**. Conjugated diene formation over time was measured at 234 nm.

When comparing the lag phases, no difference was observed between HbA and HbF. The typical lag phase was in the range of 2–4 min. However, a significant difference was observed in the propagational phase between the two Hbs. A maximum rate of conjugated diene formation was determined to be 1.31 ± 0.09 μM min^−1^ for HbA and 1.07 ± 0.06 μM min^−1^ for HbF (Figure 5). In the presence of 2 μM ascorbate, the maximum rates of diene formation were reduced to 1.24 ± 0.10 μM min^−1^ and 1.01 ± 0.13 μM min^−1^ for HbA and HbF, respectively. In addition, the lag phases were extended to 6–8 min. At a low concentration of ascorbate, the onset of reaction was thus delayed, but once the reaction was initiated, the rate of the reaction was the same as the rates without reductant.

**Figure 6:**
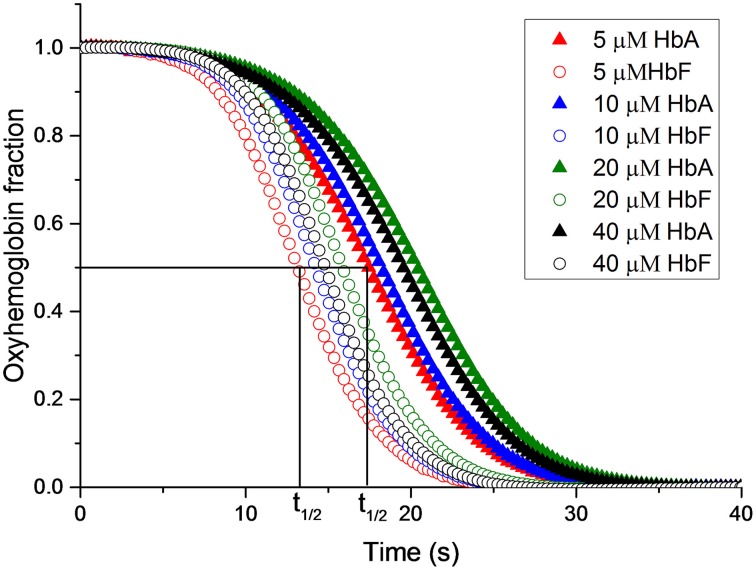
**The reaction between oxyhemoglobin and nitrite**. Different oxyHb concentrations were incubated with 2 mM NaNO_2._in 20 mM sodium phosphate buffer buffer pH 7.4 at 25°C. The increments of oxyHb levels were monitored at at 577 nm. The half-time of the reaction (t_1/2_) is indicated.

**Figure 7:**
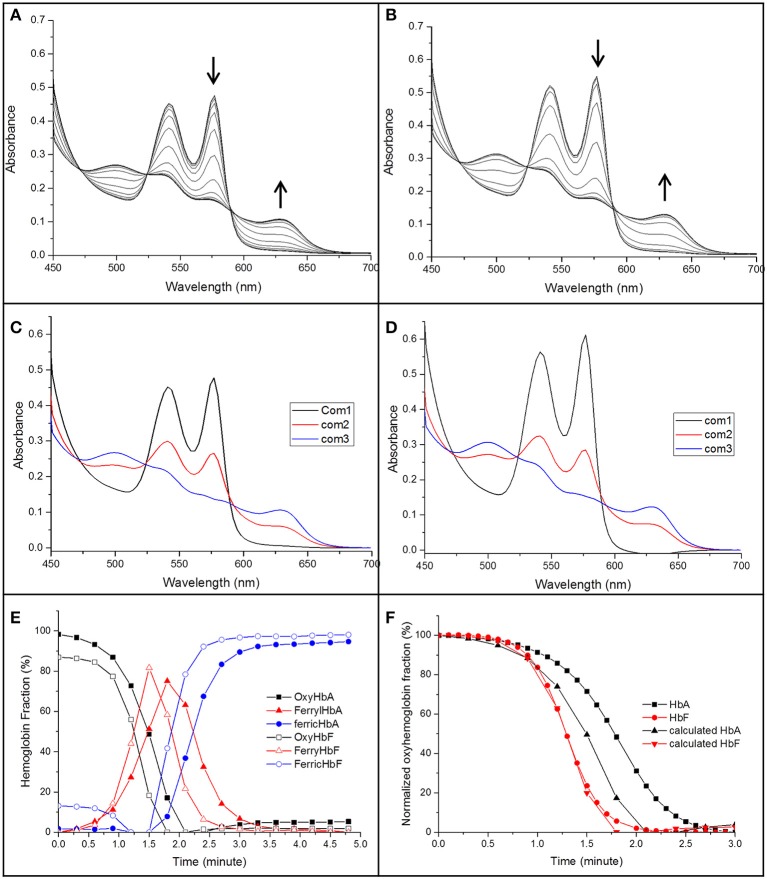
**The spectra of the reaction between 40 μM of oxyHb and 1 mM NaNO_2_ in 20 mM sodium phosphate buffer buffer pH 7.4 at 25°C (A: HbA, B: HbF)**. The spectra were recorded in the 450–700 nm region every 18 s. The decrease of oxyHb and the increase of ferric Hb during the reaction are indicated by arrows. A derived spectra component analysis from CCA plus is shown in **(C,D)** for HbA and HbF, respectively, **(E)** gives an overview when a three component analysis were prepared. **(F)** Shows a comparison between the oxyHb fraction calculated from the CCA plus program and a direct measurement of the absorbance at 577 nm.

Elevated levels of nitrite can promote the oxidation of Hb. The reaction between oxyhemoglobin and nitrite was monitored by following the decrease in absorbance at 577 nm. It was observed that HbF was oxidized to methemoglobin more rapidly than HbA with an excess of nitrite. Hb levels ranging between 5and 40 μM with 1 mM nitrite were examined and the time needed to reach 50% conversion of the oxyHb levels, was defined as a half-time reaction value, or t_1/2_, are shown in Figure 6. The t_1/2_ values were on average 35% larger for HbA compared to HbF. When following the reaction by monitoring overlaying spectra between 450 and 700 nm, more detailed information about the intermediates of the reaction could be extracted (Figure 7). After mixing with nitrite, the oxidative status of Hb was therefore analyzed over a 5-min period. For both HbA and HbF, the reaction reached a final state with typical ferric hemoglobin spectra with peaks at approximately 500, 535, 575, and 630 nm (Figures 7A,B, HbA and HbF, respectively). The spectra obtained over time were then analyzed by the CCA plus program to derive the composition of the ingoing spectral components during reaction (Figures 7C,D). The results of such an analysis are approximate, but under the conditions used, both hemoglobins generated the same final products. The first component is very similar to an oxyHb spectrum. Similarly, the final component gives an oxidized Hb spectrum. As described previously, the final products of reaction between oxyHb and nitrite are metHb and nitrate (Keszler et al., 2008). The component analysis also generated intermediate products which showed similarity with the partially oxidized Hb, showing peaks at 500 and 630 nm, and oxyHb characteristics, with peaks at 540 and 575 nm. However, no clear evidence of an isosbestic point between 575 and 600 nm was observed. This indicated that there are more than two Hb populations present during the proceedings of the reaction. This is in agreement with a previous study, which also demonstrated that the major intermediate product of this reaction is ferryl Hb (Keszler et al., 2008).

By using this analysis, the presence of each component generated during the reaction could be compared for HbA and HbF. As shown in Figure 7E, metHb was formed faster for HbF compared to HbA. When considering the reaction progress, both hemoglobins started to form the intermediate species at about the same time. However, the kinetics of the reaction was clearly different. In case of HbF, the intermediate product was increased quickly and reached a maximum of about 80% at 1.5 min, but was then fully removed after another 1.25 min. HbA exhibited a significantly slower reaction progress, both in terms of intermediate formation and final removal. During the reaction with HbA, the intermediate was thus accumulated and present over a longer time period compared with HbF. When comparing the oxyHb fraction from the component analysis program with previous experiments where the conversion was followed at 577 nm, the data from component analysis were identical to the experimental results for HbF, but smaller deviations were observed for HbA (Figure 7F). The reaction between Hb and nitrite is complex; un-identifiable Hb derivative spectra could be observed in the complex spectra. When examining the data by the CCA plus program with four component analysis, no improved fit was seen, clearly indicating that a more extensive characterization is needed to fully characterize the redox reaction.

**Figure 8:**
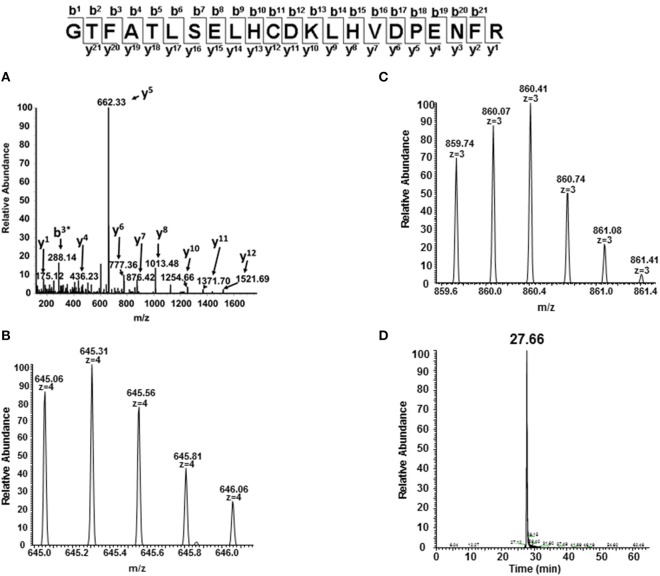
**MS/MS fragmentation spectrum and extracted ion chromatogram (XIC) of oxidized C93 tryptic peptide (residues 83–104)**. For quantitative experiments, all charged versions of Mascot identified peptides listed in Table 1 (containing the oxidized or unoxidized form) were selected in a similar manner as shown in this figure to quantify changes under different oxidative conditions. **(A)** Mascot identified MS/MS fragmentation spectrum of the oxidized C93 peptide GTFATLSELHCDKLHVDPENFR. **(B,C)** +4 and +3 charge state isotopic profiles of the oxidized C93 peptide GTFATLSELHCDKLHVDPENFR (residues 83–104). **(D)** Typical extracted ion chromatogram (XICs) for the oxidized C93 peptide (residues 83–104) generated from the ion current of the most abundant monoisotopic peak (645.31 m/z) of the +4 charge state isotopic profile listed in **(B)** XICs, were generated for all bolded peptide sequences listed in Table 1. The ratio of each oxidized “hotspot” peptide was calculated based on the sum of the XIC peak area of all forms (oxidized and unmodified) to be 100%.

In order to identify specific residues, or hotspots, in the β, γ, and α subunits of HbA and HbF that are prone to post-translational oxidation upon H_2_O_2_ exposure, quantitative mass spectrometry was utilized to quantify all hotspot containing peptide and their corresponding charge states. Extracted ion chromatograms (XICs) were generated from the most abundant monoisotopic peak of each peptide isotopic profile (Figure 8) and the resulting ratio differences were compared for oxidized and unoxidized hotspot peptides (Table 1). For comparative purposes, we focused on residues that were conserved between γ, β, and α subunits for HbF and HbA. XICs were therefore generated for the bolded peptide sequences in Table 1 representing conserved amino acids where significant differences in H_2_O_2_ induced oxidation were observed. For example, C93 is conserved in both γ and β subunits; the extent of H_2_O_2_ induced cysteine tri-oxidation was therefore monitored to identify how ferric HbA and HbF differ in their response to oxidative conditions. When analyzing the hotspot data shown in Table 2, it is clear that β subunits are more susceptible to oxidative changes than γ subunits. These data showed substantial increases in H_2_O_2_ induced oxidation of β C93 containing peptides for HbA compared to γ C93 for HbF; for 300, 400, and 500 μM there was a ~20 fold higher ratio of oxidized C93 for HbA vs. HbF. This suggests that the γ subunit is considerably more stable or resistant to oxidation than the β subunit of HbA. It should also be pointed out that HbA α subunits were slightly more oxidized than HbF α subunits, although α oxidation (as observed for C104) for both hemoglobins was considerably low. This suggests that γ may impose some level of additional stability in α subunits. Additionally, a higher level of nitrated β Y35 containing peptides for HbA compared to γ Y35 for HbF (~30 fold higher) was observed, which clearly supports the first data set indicating higher γ subunit stability.

**Table 2:** **Oxidative ratios for C93 trioxidation of 10 μM ferric HbA and HbF in the presence of H_2_O_2_ as well as nitration of 40 μM ferrous HbA and HbF in the presence of sodium nitrite**.

**Reaction condition**	**C93 oxidation %HbA β**	**C93 oxidation %HbF γ**	**C104 oxidation %HbA α**	**C104 oxidation %HbF α**
no H202	Trace	Below detection	0.4 ± 0.1%	0.5 ± 0.1%
100 μM H202	32 ± 2.8%	1.4 ± 0.4%	0.7 ± 0.2%	1.3 ± 0.2%
200 μM H202	43 ± 2.9%	1.1 ± 0.5%	0.9 ± 0.1%	0.3 ± 0.2%
300 μM H202	45 ± 3.3%	2.0 ± 0.4%	1.3 ± 0.2%	0.9 ± 0.3%
400 μM H202	52 ± 7.0%	2.6 ± 0.9%	3.7 ± 0.3%	0.5 ± 0.1%
500 μM H202	46 ± 4.4%	2.2 ± 0.9%	4.2 ± 03%	0.4 ± 0.01%
**Reaction condition**	**Tyr 35 nitration %HbA β**	**Tyr 35 Nitration %HbF γ**	**Tyr24 oxidation %HbA α**	**Tyr24 oxidation %HbF α**
40 μM ferrous HbA/HbF 1 mM NaNO2	3.2 ± 0.1%	0.1 ± 0.01%	0.3 ± 0.04%	0.02% ± 0.01

## Discussion

The overall three-dimensional structure of HbF is very similar to HbA; however, differences in 39 amino acids of the gamma chain influence the physical and chemical properties of HbF. The major part of variation compared to HbA is found in the N-terminal A helix where the γ subunit differs from the β-subunit in 8 of 18 amino acid residues (Frier and Perutz, 1977). The role of the A helix is to maintain the tetrameric integrity of the Hb molecule, which is 70-fold stronger compared to HbA (Dumoulin et al., 1997; Yagami et al., 2002). The reduced tendency of HbF to dissociate into dimers has modified biophysical properties, and significant physiologic consequences. For instance, fetal red cells show enhanced resistance to the malaria parasite (Shear et al., 1998). Other substitutions, like the Glu43Asp replacement, which is located at the allosteric interface, together with the substitution of His143Ser at the DPG binding site, are critical in increasing oxygen affinity and facilitating a relevant physiological oxygen transfer from maternal to fetal blood (Chen et al., 2000). These substitutions could be part of a functional adaptation of Hb to allow its activity under conditions with lower oxygen levels. The fetus is largely developed under hypoxic conditions. Exposure to higher O_2_ concentrations at a later developmental stage or at childbirth, may lead to an escalation of oxidative stress levels. The presence of additional H_2_O_2_ during exposure to elevated oxygen levels can in turn trigger antioxidant enzyme systems, including catalase and glutathione peroxidase. However, Hb also exhibits an intrinsic peroxidase activity. This implies that Hb has a protective role against H_2_O_2_ which has been demonstrated in cell culture systems under oxidative stress (Widmer et al., 2009). As shown in this study, HbF carries a higher peroxidase activity which facilitates the removal of H_2_O_2_. Therefore, this activity may shield the fetus, both under normal physiological conditions and when impairment in the protective antioxidant enzyme systems has occurred.

Under normal physiological conditions, approximately 3% of Hb undergoes autoxidation to produce ferric Hb. This process is accompanied by a release of O^•−^_2_ which rapidly dismutases to H_2_O_2_ by superoxide dismutase (SOD) (Johnson et al., 2005). The level of H_2_O_2_ in red blood cells is controlled by catalase and the concentration of H_2_O_2_ is relatively low, approximately 2 × 10^−10^ M (Giulivi et al., 1994). The H_2_O_2_ concentration in normal plasma is 4–5 μM (Nagababu and Rifkind, 2000), however, these levels can increase as a result of a tissue injury, or under oxidative stress conditions. H_2_O_2_ is a reactive oxygen species which can cause damage to cells and tissues. Hb is also susceptible to H_2_O_2_ oxidation. The intermediate species of this reaction, ferryl Hb, is considered to be as a very reactive species, however, the formed ferryl Hb decays rapidly, either through a comproportionation reaction with ferrous heme or through autoreduction to reform ferric Hb (Giulivi and Davies, 1990).

The oxidation of Hb by hydrogen peroxide can induce structural modifications on the globin molecule (Jia et al., 2007). Mass spectrometric approaches have thus previously revealed that cysteine amino acids are extensively oxidized to cysteic acid. Moreover, oxidation of these residues leads to a partial collapse of the β-chain. Effects of H_2_O_2_ on the α-chain have also been observed, including the covalent linkage of the heme group to Ser-138. In this study, all identified peptides after treating with H_2_O_2_ are consistent with previous studies except at position 112 on the γ-chain of HbF which is substituted by threonine (Thr) and is thereby inert to H_2_O_2_ attack. The amino acids susceptible to H_2_O_2_ in the β/γ chain (Cys and Met) are on the surface and are constantly exposed. In HbF, Thr112 is located on the α1γ1 interface. Modification of this residue could disturb subunit interactions within the Hb dimer leading to quaternary structural changes. However, after exposure to H_2_O_2_, this amino acid is not modified and the α1γ1 contact is still intact. This renders HbF more stable compared to HbA. The peroxidase activity of ferric Hb and H_2_O_2_ results in the formation of higher oxidation species like ferryl Hb where the free radical may reside on β Tyr -145 (Cooper et al., 2012). The stable HbF tetramer could prevent the intermolecular leakage of newly formed free radicals. Moreover, the distance from the center heme in the γ chain to Tyr 145 is about 11Å; this distance is within the range for electron transfer (Moser et al., 1992) and it then can be reused in a catalytic cycle. This might be the mechanism responsible for a higher autoreduction rate found in HbF.

Both γ and β subunits share a common evolutionary ancestry, transcriptional control and thermodynamically exhibit a higher redox potential than α subunits (Strader et al., 2014). In fact, recent proteomic and crystal structures studies indicate that human α subunits from both wildtype and mutant Hb accumulate much smaller amounts of ferryl and ferryl protein radicals than γ and β subunits when exposed to H_2_O_2_ in solution (Strader et al., 2014). In this study, a fetal γ-globin mutation (γ67(E11)Val→Met) in which Met67 was post-translationally oxidized to aspartic acid (Asp) in blood from a newly born child, known as Hb Toms River. Under similar experimental oxidative conditions, (in the presence of increasing H_2_O_2_), the conversion of Met→Asp at position 67 in the β subunits of recombinant adult Bristol Hb (β 67(E11)Val→Met), was also observed but not in the structurally equivalent α subunit variant (Hb Evans) (α62(E11) Val→Met). These findings confirm that a post-translational oxidative modification occurs within the redox active γ or β but not within α subunits of human Hb and correlate with the apparent differences in redox reactivity of HbF and HbA reported in this work.

The reaction between oxyHb and nitrite supports the hypothesis that HbF has a faster turnover rate. The reaction between oxyHb and nitrite starts slowly and H_2_O_2_ and metHb are produced in this step. The reaction is then accelerated by the fast conversion of metHb by the newly formed H_2_O_2_ generating ferryl Hb as an intermediate species. Ferryl Hb, however, can be reduced quickly with nitrite to yield a metHb and a nitrite radical which then can react directly with oxyHb to generate nitrate. The overall reaction hence produces metHb and nitrate. In HbF, the intermediate species of the reaction, ferryl Hb, is converted back more quickly to metHb than HbA thereby preventing an accumulation of the more toxic ferryl Hb in the system. In human red blood cells, metHb is then converted to ferrous Hb by methemoglobin reductase. The lower lipid oxidation rate observed for the HbF reaction in the liposome oxidation study also supports that a faster ferric-ferryl Hb redox cycle could be a benefit especially under a reducing environment.

In summary, we propose that having different Hb molecules in each stage of fetal development is not only an adaptation for efficient oxygen transfer to the fetus, but it also involves a protection against reactive oxygen species. Moreover, the ability of HbF to remove H_2_O_2_ faster compared to HbA, which together with a higher structural stability could be used as strategies for an improved design of HBOCs. A previous study also suggests that deoxy HbF exhibits a higher nitrite reductase activity (Blood et al., 2009). Therefore, it can produce NO in the presence of nitrite. As suggested earlier, the toxicity of Hb can be further ameliorated by modulating the electron transfer pathway to enhance removal of the toxic ferryl Hb (Reeder et al., 2008a). HbF may be particularly useful as starting material for HBOC applications, since it produces lower levels of ferryl Hb, and the ferryl form can be readily reduced back to ferric Hb by antioxidants found in human circulation.

### Conflict of interest statement

The authors declare that the research was conducted in the absence of any commercial or financial relationships that could be construed as a potential conflict of interest.
